# Differential Induction of Long-Term Potentiation in the Horizontal versus Columnar Superficial Connections to Layer II Cells of the Entorhinal Cortex

**DOI:** 10.1155/2008/814815

**Published:** 2008-06-30

**Authors:** Li Ma, Angel Alonso, Clayton T. Dickson

**Affiliations:** ^1^Department of Neurology and Neurosurgery, McGill University and Montreal Neurological Institute, Montreal, PQ, Canada H3A 2B4; ^2^Department of Psychology, University of Alberta, Edmonton, AB, Canada T6G 2E9; ^3^Department of Physiology, University of Alberta, Edmonton, AB, Canada T6G 2H7; ^4^Centre for Neuroscience, University of Alberta, Edmonton, AB, Canada T6G 2H7

## Abstract

The entorhinal cortex (EC) is a nodal and independent mnemonic element of the medial temporal lobe memory circuit as it forms a bidirectional interface between the neocortex and hippocampus. Within the EC, intra- and inter-lamellar associational connections occur via horizontal and columnar projections, respectively. We undertook a comparative study of these two inputs as they converge upon EC layer II cells using whole-cell patch techniques in an adult rat EC horizontal slice preparation in which the deepest layers (V-VI) had been dissected out. Electrical stimulation of layers I and III during GABA blockade allowed us to study excitatory synaptic properties and plasticity in the horizontal and columnar fibre systems, respectively. Both pathways exhibited AMPA- and NMDA-receptor mediated transmission and both exhibited long-term potentiation (LTP) after high-frequency (tetanic) stimulation. LTP in the horizontal, but not in the columnar pathway, was blocked by NMDA receptor antagonism. Intriguingly, LTP in both appeared to be mediated by post synaptic increases in Ca^2+^ that may be coupled to differing second messenger pathways. Thus, the superficial excitatory horizontal and columnar associative pathways to layer II have divergent mechanisms for LTP which may endow the EC with even more complex and dynamic processing characteristics than previously thought.

## 1. INTRODUCTION

The entorhinal cortex (EC) is a prominent component of the medial temporal lobe memory system. The superficial layers (II and III) of the EC receive an extensive input from multimodal sensory associational areas of neocortex and, in turn, project to all subregions of the hippocampal formation. Output stations of the hippocampus, including CA1 and subiculum, project back to the deep layers of entorhinal cortex (VI & V), reciprocating the input channels [1, 2]. Therefore, the EC serves as a bidirectional interface between the neocortex and hippocampal formation and as such forms a nodal part of the cortico-hippocampo-cortical loop that is the brain's hardware for the formation of declarative memories.

The importance of the EC in memory processing, however, is thought to go beyond just its interconnections with the hippocampus. One suggestion is that the EC and other parahippocampal regions serve as a temporary memory store that is critical to normal hippocampal-dependent memory processing [3]. Behavioral studies have suggested that lesions involving the EC are followed by learning and memory deficits in mammals (reviewed in [4]). Indeed, early stage tissue from Alzheimer's patients in which memory impairments are just subclinical demonstrates neurodegeneration in the superficial layers of the EC specifically [5, 6]. Still other work has shown that embryonic entorhinal transplants partially ameliorate the deficits in spatial memory in adult rats with EC lesions [7]. Perhaps the strongest evidence for an independent role of the EC in memory is that a number of entorhinal cells demonstrate persistent activity in the delay phase of memory tasks which correlates to the retention of information necessary to perform during a subsequent go phase [8, 9]. Indeed, entorhinal cells also demonstrate intrinsic “memory-like” persistent firing properties dependent upon associative convergence of excitatory inputs and cholinergic neuromodulation [10].

Synaptic plasticity of excitatory glutamatergic responses, via long-term potentiation (LTP), has been proposed as a mechanism underlying learning and memory. The most commonly studied form involves an NMDA-receptor dependent process whereby postsynaptic Ca^2+^ influx through this ligand-gated channel induces changes via a series of intracellular second messengers (typically beginning with the calcium/calmodulin-dependent kinase: CaMKII) that result in the enhancement of neurotransmission [11].It has also been shown that LTP can occur through non-NMDA dependent triggers such as activation of either voltage-dependent calcium channels or metabotropic glutamatergic receptors which can also lead to increases in Ca^2+^ influx and LTP via potentially overlapping intracellular mechanisms [12–14]. In addition, non-CaMKII-dependent processes have also been elucidated [11, 15, 16]. Although perhaps differing in their cellular induction mechanisms, all of the above are thought to express their effects mainly through postsynaptic changes to AMPA-type receptors that result in an enhancement of glutamate responsiveness. Changes to presynaptic release (due to growth of new contacts and enhancement of release machinery) have also been suggested to play a role (reviewed in [11, 15]).

A different form of LTP exists that appears to involve a completely different induction and expression mechanisms. This form, first elucidated in the mossy fibre input to CA3 pyramidal neurons in the hippocampus, does not require either NMDA receptor activation or an increase in postsynaptic Ca^2+^ [17]. The locus of induction and expression of this form of LTP is presynaptic [18–20], involving no changes in postsynaptic receptivity. This presumed increase in neurotransmitter release is accompanied by a marked and long-lasting decrease in the paired-pulse facilitation ratio [18].

The cellular mechanisms underlying LTP in the EC remain understudied. Field recordings of LTP phenomenon have been reported in layer II and some interesting differences between deep and superficial associational pathways have been reported [21, 22]. To date, no studies have assessed, in the same superficial layer II cells, the differential properties of LTP in these two important pathways. Here, using whole-cell-recording technique, we investigated the physiology, pharmacology, and plasticity of both horizontal and columnar associative inputs to layer II cells using whole cell techniques in an in vitro slice preparation. These results have been previously published in abstract form [23].

## 2. MATERIALS AND METHODS

### 2.1. General

Brain slices were prepared from male Long-Evans rats (100–200 g) using standard procedures [24]. All methods used conformed to the guidelines established by the Canadian Council on Animal Care and the Society for Neuroscience. Animals were quickly decapitated, and the brain was rapidly removed, blocked, and placed in a cold (4–6°C) oxygenated normal Ringer's solution containing (in mM): 124 NaCl, 5 KCl. 1.3 NaH_2_PO_4_, 2.0 CaCl_2_, 2.0 MgSO_4_, 26 NaHCO_3_, and 10 glucose (pH 7.4, by gaseous saturation with 95% O_2_ & 5% CO_2_). Horizontal slices from the retrohippocampal region were cut at a thickness of 400 *μ*m using a vibratome (Pelco, Redding, Calif, USA). After at least an hour recovery period during which they were submerged in normal Ringer's at room temperature (23°C–25°C), individual slices were transferred to a recording chamber located on the stage of an upright, fixed-stage microscope (Axioskop, Zeiss). Slices were submerged and perfused continuously with a saturated (95% O_2_ + 5% CO_2_) solution containing (in mM) 126 NaCl, 2.5 KCl, 1.3 NaH_2_PO_4_, 2.4 CaCl_2_, 1.4 MgSO_4_, 26 NaHCO_3_, 10 glucose, and 0.05 picrotoxin (a GABA_A_ receptor antagonist) at room temperature. Before being transferred, a cut was made between layer III and layer V regions of entorhinal cortex to prevent the propagation of epileptiform activity. The microscope was equipped with a water immersion objective (40–63X: long-working distance), Nomarski optics, and a near-infrared charge-coupled device (CCD) camera (Sony XC-75). With this equipment, individual cells in layer II could be visualized via video microscopy and targeted for recording [25].

### 2.2. Recording

Somatic whole-cell voltage-clamp recordings [26] were made under visual control using 4–7 MΩ electrodes. The whole-cell solution contained (in mM): 130 K-Gluconate, 5 NaCl, 2 MgCl_2_, 10 Hepes, 0.5 EGTA, 2 ATP, 0.4 GTP (pH 7.2–7.4 with 1 M KOH; osmolarity: 290–300 mOsm). In experiments designed to chelate intracellular Ca^2+^, 10 mM BAPTA was substituted for equimolar K-Gluconate. To investigate properties of evoked EPSCs at varying membrane potentials, 130 mM Cs-Gluconate and 0.8 mM QX314-Cl were used in the whole-cell solution in place of K-Gluconate. The liquid junction potential was estimated using the method of Neher (1992) and was found to be 8 mV. No correction of transmembrane potential was applied for the liquid junction potential unless indicated otherwise.

Whole cell voltage clamp recordings were made using an Axoclamp2A or Axopatch200B amplifier (Axon Instruments, Foster City, Calif, USA). The low-pass filter (−3 dB) was set to 1 kHz, and the resulting current trace was digitized to computer at 2 kHz using a Digidata 1320 A-D converter driven by Pclamp7 (Axon Instruments).

Cells were held at −60 to −70 mV during recordings unless indicated otherwise. Series resistance was measured automatically using Pclamp7 software and those with values higher than 30 MΩ in whole-cell configuration were discarded. In the remainder, series resistance was compensated >40% with the amplifier's built-in compensation circuitry. Input resistance of cells (averaging 169.67 ± 5.67 MΩ, *N* = 171) was calculated by measuring current deflections in response to 5 mV hyperpolarizing voltage pulses from the holding potential and was monitored through each recording. A short-duration hyperpolarizing voltage step (−5 mV, 250 millisecods) was always applied to each sweep just prior to stimulation to monitor any change in input resistance and series resistance. Cells exhibiting detectible changes in the elicited current process to the hyperpolarization during recording were discarded.

### 2.3. Stimulation and experimental protocol

**Figure 1:**
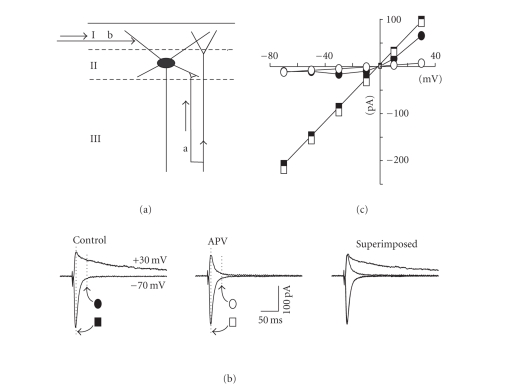
Properties of evoked EPSCs in EC layer II neurons. (a) Schematic diagram of the ascending columnar (arrow a) and the horizontal (arrow b) inputs converging on a layer II cell in entorhinal cortex (I, II, and III refer to the superficial layers of the EC). (b) Stimulation of layer III evoked excitatory postsynaptic currents that had different profiles at hyperpolarized (−70 mV) versus depolarized (+30 mV) potentials and sensitivity to APV. For these experiments, 130 mM CsGluconate and 0.8 mM QX314-Cl were substituted for KGluconate in the recording pipette. Corrections were made for the liquid junction potential. In control conditions (leftmost panel), EPSCs demonstrated only a fast decay component at −70 mV, but showed a slower decay component at +30 mV. In the presence of 50 *μ*M DL-APV (middle panel), only the fast component was observed at both holding potentials (a direct comparison is shown in the superimposed traces in the rightmost panel). (c) I-V relationship for EPSC components measured at peak and 30 milliseconds post peak for the same cell (see dotted lines in (b)). The peak amplitude (closed square plots) showed a linear relationship across the full range of membrane potentials tested (−70 to +30 mV). This was unchanged following application of APV (open square plots). In control conditions, the amplitude measured 30 milliseconds following the peak (filled circles) was lower and showed a negative slope region at depolarized membrane potentials levels (−60 to −20 mV) but became nearly linear thereafter. In the presence of APV (open circles), the negative slope region was abolished and the resultant plot was linear across the full range of membrane potentials.

Electrical stimulation of afferent pathways to layer II was conducted through bipolar, stainless steel electrodes insulated except at their tips. These were positioned on the surface of the slice in layers I/II or III (see diagram in Figure 1(a)). Afferent inputs were activated in this way by passing short (100 *μ*s) current pulses of 0.5–4.0 mA using a pulse generator (PG-4000 Cygnus Tech, NY) coupled to a stimulus isolation/constant current generator (Model A395 WPI). EPSCs were evoked once every 30 seconds, and the basal amplitude of EPSCs was set to 100–200 pA by adjusting the stimulation intensity. EPSCs were accepted as being monosynaptic if they exhibited short and consistent latencies that did not change with increasing stimulus intensity.

Following at least 5 minutes of stable baseline recording, LTP was elicited using a high-frequency (tetanic) stimulation train (HFS: 100 Hz; 1 second) at the same intensity used for baseline recording. Typically, HFS was delivered no more than approximately 15 minutes following the formation of whole cell configuration. Little to no potentiation was observed if HFS was applied at times greater than 30 minutes after achieving whole cell mode. Successful LTP was evaluated statistically in comparison to baseline values at a time point 30 minutes following HFS for each experiment.

Paired pulse facilitation protocols were used in order to assess any changes in presynaptic release mechanisms. The two pathways were stimulated either singly or alternatively at short intervals (50–70 milliseconds). PPF ratio was computed by dividing the peak amplitude of the second EPSC by the peak amplitude of the first in each experiment.

Application of drugs was conducted by adding stock solutions directly to the superfusion medium to make appropriate final concentrations. DL-APV (Tocris) and CNQX (Tocris) were made up as a 10 mM stock solution in an equivalent volume mix of 1M NaOH and dimethyl sulfoxide. These were pipetted at smaller volumes into centrifuge tubes and stored frozen (−20°) until used. Picrotoxin and BAPTA were purchased from “Sigma (St. Louis, Mo).”

### 2.4. Data analysis

The amplitude of EPSCs was measured offline using Clampfit 6 (Axon Instruments), by calculating the difference between the peak deflections relative to the average holding current level computed for the 10 milliseconds preceding the stimulus. Average traces as shown in figure insets represented means of 5–10 individual EPSC sweeps. Peak amplitudes or paired-pulse facilitation (PPF) ratios were computed from these averages. Potentiation of EPSC amplitudes or PPF ratios following the application of HFS was expressed by normalizing amplitudes to the baseline values pre-HFS. Reported values reflect arithmetic means ± standard error of the mean (SEM), and statistical significance was determined by paired and unpaired Student's *t*-tests.

## 3. RESULTS

### 3.1. Characterization of EPSCs in layer II cells

Application of electrical stimulation to either lateral positions in layers I/II or deep positions in layer III evoked pure excitatory postsynaptic currents (EPSCs) in layer II cells under our recording and superfusion conditions (with added 50 *μ*M picrotoxin). These EPSCs were routinely characterized as monosynaptic but occasionally polysynaptic or even antidromic responses were observed. In these latter cases, the stimulation electrode was physically adjusted or even moved on the surface of the slice until it evoked a pure monosynaptic response. The latency of responses was 4.01 ± 0.13 milliseconds (layer I stimulation) and 3.85 ± 0.11 milliseconds (layer III stimulation). These responses were completely blocked by TTX (1 *μ*M), cadmium (200 *μ*M), and the nonspecific glutamate antagonist, kynurenic acid (0.5 mM) (not shown).

To characterize the basic properties of the evoked EPSCs, we recorded the response over a broad range of membrane potentials (see Figures 1(b), 1(c)—layer III stimulation in this case). EPSCs displayed only a fast decay component when the membrane potential was held at −70 mV. A slow decay component became apparent as the holding potential was depolarized (see Figure 1(b), *n* = 6). These fast and slow decay components of EPSC appeared similar to AMPA- and NMDA-receptor mediated currents in other parts of the central nervous system. In order to examine this possibility in further detail, we constructed the current-voltage (I-V) relation of the EPSC response measured at the peak and 30 milliseconds after the peak (see dashed lines in Figure 1(b)). The peak I-V relation (filled squares in Figure 1(c)) was linear over the entire voltage range with a reversal potential of 0 mV whereas the I-V relation measured 30 milliseconds after the peak (filled circles in Figure 1(c)) was nonlinear with a region of negative slope resistance in the range of −60 to −20 mV. This nonlinear I-V behavior is typical for NMDA-receptor mediated EPSCs. Indeed, bath application of the NMDA receptor antagonist DL-APV (50 *μ*M) abolished the slow decaying component of the EPSC at depolarized potentials without any significant effect on the early component (see Figures 1(b), 1(c)). In consequence, in the presence of DL-APV the peak I-V relation remained unchanged (open squares in Figure 1(c)). A small synaptic current could still be measured at 30 milliseconds but this displayed a completely linear I-V relation (open circles in Figure 1(c)) and was thus due to the late phase of the fast EPSC. In contrast to DL-APV, the selective non-NMDA receptor blocker CNQX (10 *μ*M) completely abolished the EPSC at hyperpolarized potentials (not shown). These data suggest that the properties of EPSCs evoked by stimulation of both horizontal and columnar afferents in EC layer II cells are similar to their glutamatergic counterparts in the hippocampus, or indeed, in other parts of the central nervous system.

### 3.2. Independence of the horizontal and vertical associational pathways

**Figure 2:**
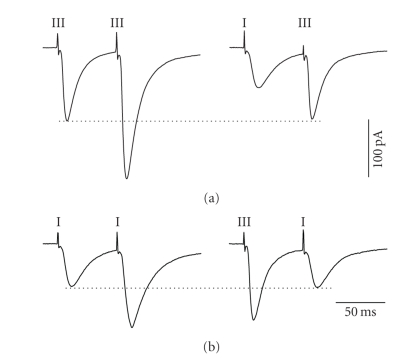
Afferent independence between the ascending columnar and horizontal associational pathways. The two pathways were stimulated repetitively (left panels) or alternatively (right panels) using a short interpulse interval (60 milliseconds). Each trace represents an average of 16 sweeps. Although paired-pulse facilitation (PPF) was observed in both (a) the ascending and (b) horizontal pathways when stimulated independently (right panels), no facilitation was observed when stimulating each alternatively (left panels). Dotted lines indicate the maximum amplitude to the first pulse in each case.

In order to assure that our stimulation conditions evoked activity in independent sets of inputs converging in layer II, we used homosynaptic and heterosynaptic paired pulse facilitation (PPF) protocols. The two pathways were stimulated independently or alternatively at short intervals (50–70 milliseconds) and we measured whether the amplitude of EPSCs evoked by the second stimulation pulse was increased following the first. Since PPF reflects an increase in neurotransmitter release based on prior activity in the same synapse, any facilitation observed across different site stimulation would reflect the activation of overlapping synapses. Consistent with this idea, each pathway could independently demonstrate PPF (see Figures 2(a), 2(b): left panels). In contrast, heterosynaptic stimulation protocols (either layer I followed by layer III or vice versa) failed to show any PPF (see Figures 2(a), 2(b): right panels). Using the same protocol in a total of three cells, we obtained similar results which indicated that the two converging pathways were indeed independent.

### 3.3. LTP of the horizontal associational pathway

**Figure 3:**
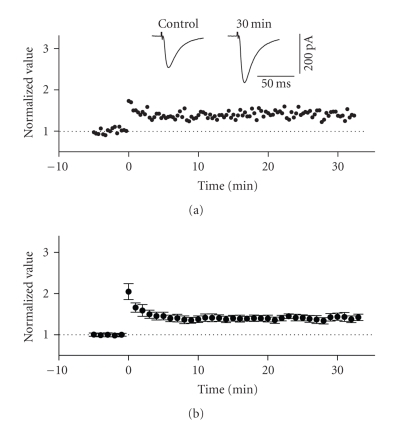
LTP in the horizontal associative pathway of the superficial entorhinal cortex. The stimulation electrode was positioned on the surface of layer I, lateral to the recording electrode. (a) Normalized scaled amplitude of peak EPSCs evoked by test pulses in a layer II cell is plotted against time. HFS at time zero led to an immediate and long-lasting potentiation of EPSCs. The inset shows two traces reflecting averaged EPSCs at baseline (pre-HFS) and at 30 minutes post-HFS. (b) Average normalized scaled amplitude of peak EPSCs across 15 cells. The value at 30 minutes post-HFS (1.401 ± 0.087) is significantly different from baseline (*P* < .01).

Previous work using both field and intracellular methods have demonstrated that the inputs to layer II activated by stimulation of layer I (which include horizontal association fibres from layer II and extraentorhinal afferent fibre systems) exhibit homosynaptic LTP [21, 27]. We confirmed this in our own whole cell recording conditions using HFS protocols. Posttetanic potentiation (PTP) was observed in all recorded cells following the application of high-frequency stimulation (100 Hz, 1 second) to layer I at sites lateral to the recording electrode position (see Figure 3(a)). LTP of EPSCs following PTP was observed in 65% of cells tested (15 of 23) and lasted for more than 30 minutes following HFS. The average normalized value of peak EPSCs in these cells increased by a factor of 1.401 ± 0.087 of baseline values at 30 minutes following HFS (see Figure 3(b)). This value was significantly different from baseline (*n* = 15, *P* < .01).

**Figure 4:**
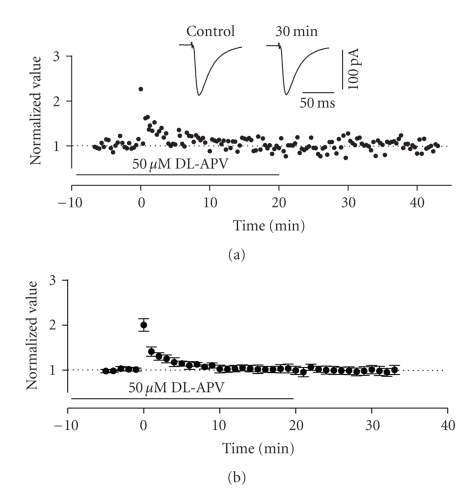
Induction of LTP in the horizontal pathway required activation of NMDA-receptors. (a) Normalized scaled amplitude of peak EPSCs evoked by test pulses in a layer II cell is plotted against time. Time point of application of 50 *μ*M DL-APV is indicated by the line. HFS at time zero led to short-term (PTP) but not long-term potentiation. The inset shows two traces reflecting averaged EPSCs at baseline (pre-HFS) and at 30 minutes post-HFS. (b) Average normalized scaled amplitude of peak EPSCs across 6 cells in the presence of APV as indicated by the line. The value at 30 minutes post-HFS (0.979 ± 0.095) was not significantly different from baseline (*P* > .05).

To determine whether induction of LTP in this pathway required NMDA receptor activation, we tested slices treated with the NMDA receptor antagonist DL-APV (50 *μ*M). Under these conditions, HFS to lateral sites in layer I only produced a short term (PTP) facilitation of EPSCs in all cells tested. These values returned to baseline levels within 10 minutes following HFS (see Figure 4(a)). The average normalized value of peak EPSCs was 0.979 ± 0.095 at 30 minutes after HFS in the presence of APV which was not significantly different from baseline values (*n* = 6, *P* > .01, see Figure 4(b)).

**Figure 5:**
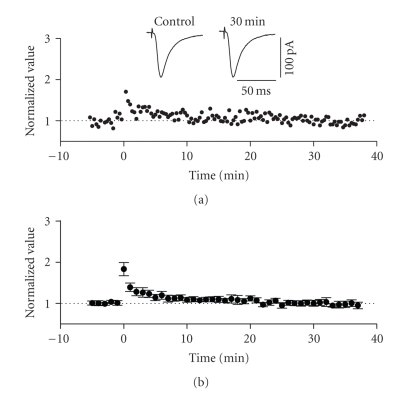
Induction of LTP in the horizontal pathway required an increase in postsynaptic calcium. (a) Normalized scaled amplitude of peak EPSCs evoked by test pulses in a layer II cell loaded with 10 mM BAPTA is plotted against time. HFS was routinely applied about 15 minutes after formation of whole-cell mode to ensure adequate BAPTA diffusion. The inset shows two traces reflecting averaged EPSCs at baseline (pre-HFS) and at 30 minutes post-HFS. (b) Average normalized scaled amplitude of peak EPSCs across 5 BAPTA-loaded cells. HFS at time zero induced only short-term (PTP) but not long-term potentiation. The value at 30 minutes post-HFS (1.008 ± 0.068) was not significantly different from baseline (*P* > .05).

To determine if HFS-induced LTP was also dependent upon a rise in postsynaptic Ca^2+^, we tested the effects of dialysing the fast chelator BAPTA (10 mM) into the postsynaptic cell. As in NMDA blockade conditions, HFS of lateral layer I led to a short term facilitation of EPSCs only. This facilitation returned to baseline levels in slightly less than 10 minutes following the application of HFS (see Figure 5(a)). The average increase in the amplitude of EPSCs at 30 minutes post-HFS (1.008 ± 0.068) was not significantly different from baseline (*n* = 5, *P* > .01, see Figure 5(b)).

**Figure 6:**
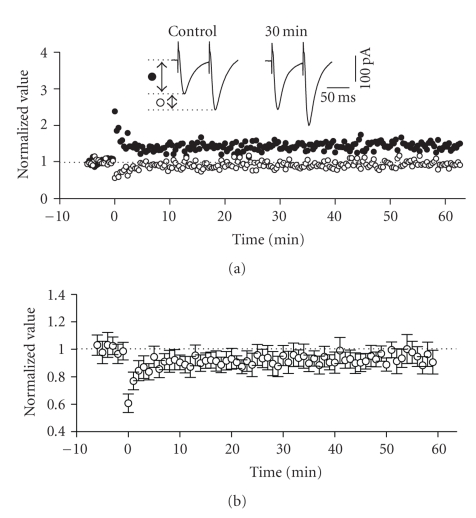
Expression of LTP in the horizontal pathway was associated with a slight change in paired-pulse facilitation (PPF) ratio. (a) Normalized scaled amplitude of the peak EPSCs evoked by the first of a pair of test pulses separated by 60 milliseconds (filled circles) and the normalized paired pulse facilitation ratio (open circles) in a layer II cell is plotted against time. The inset shows two traces reflecting averaged paired EPSCs at baseline (pre-HFS) and at 30 minutes post-HFS. PPF ratio was calculated as the amplitude of the peak of the second EPSC divided by the first. (b) Average normalized scaled amplitude of PPF ratio in 10 cells. HFS at time zero induced a short-term decrease which decayed back to near baseline levels. The values at 30 and 50 minutes after HFS, however, (0.944 ± 0.065 and 0.966 ± 0.068, resp.) were significantly different from baseline (*P* < .01).

In order to assess if the expression of LTP in the horizontal associative pathway was solely dependent on postsynaptic mechanisms, we investigated any changes in PPF ratio. HFS to lateral layer I led to an immediate and obvious decrease in PPF which soon returned to stable (and near-baseline) levels within 5 minutes (see Figure 6(a)). PPF ratio at 30 and 50 minutes post-HFS was 0.917 ± 0.084 and 0.938 ± 0.062, respectively, which were both slightly, but significantly, less than baseline (*n* = 8, *P* < .01, see Figure 6(b)). Thus, while the primary component of LTP in this pathway appeared to be postsynaptic in expression, a small component also appeared to involve a presynaptic locus.

### 3.4. LTP of the ascending columnar associational pathway

**Figure 7:**
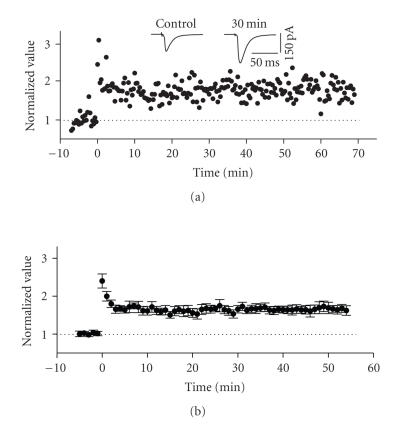
LTP in the columnar associative (ascending) pathway of superficial entorhinal cortex. The stimulation electrode was positioned on the surface of layer III, just deep to the recording electrode. (a) Normalized scaled amplitude of peak EPSCs evoked by test pulses in a layer II cell is plotted against time. Application of HFS delivered at time zero led to an immediate and long-lasting increase in the amplitude of EPSCs. This posttetanic potentiation lasted less than 5 minutes before it reached a stable and potentiated level. The inset shows two traces reflecting averaged EPSCs at baseline (pre-HFS) and at 30 minutes post-HFS. (b) Average normalized scaled amplitude of peak EPSCs across 11 cells. The values at 30 and 50 minutes post-HFS (1.65 ± 0.102 and 1.68 ± 0.128, resp.) were significantly different from baseline (*P* < .01).

Application of HFS to afferent fibres in layer III caused an immediate and long-lasting potentiation of EPSCs in 79% of cells tested (23 of 29) which lasted for the duration of the recording situation (>30 minutes post-HFS). Short-term posttetanic potentiation (PTP) was a consistent feature of this facilitation and lasted for just less than 5 minutes following HFS (see Figure 7(a)). The average normalized value of peak EPSCs increased by a factor of 1.65 ± 0.102 and 1.68 ± 0.128 of baseline (pre-HFS values) at 30 and 50 minutes, respectively, following HFS (see Figure 7(b)). Both values were significantly different from baseline (*n* = 23, *P* < .01).

**Figure 8:**
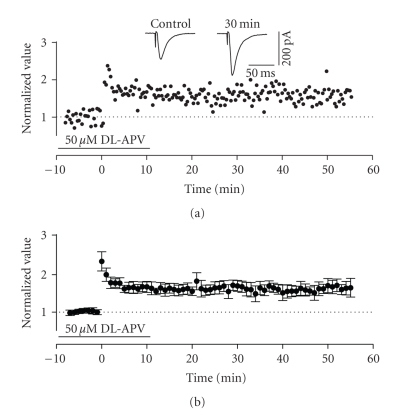
Induction of LTP in the ascending pathway did not require activation of NMDA-receptors. (a) Normalized scaled amplitude of peak EPSCs evoked by test pulses in a layer II cell is plotted against time. Time point of application of 50 *μ*M DL-APV is indicated by the line (note, all experiments involved superfusions of APV for more than 10 minutes before application of HFS at time zero). HFS led to both short-term (PTP) and long-term potentiation. The inset shows two traces reflecting averaged EPSCs at baseline (pre-HFS) and at 30 minutes post-HFS. (b) Average normalized scaled amplitude of peak EPSCs across 10 cells in the presence of APV as indicated by the line. The value 30 and 50 minutes after was 1.695 ± 0.145 and 1.707 ± 0.19, respectively, which were both significantly different from baseline values (*P* < .01).

To determine whether induction of LTP in this pathway required NMDA receptor activation, we tested slices treated with the NMDA receptor antagonist DL-APV (50 *μ*M). Surprisingly, both the induction and maintenance of LTP appeared unaffected by this manipulation even though slices were superfused for at least 10 minutes prior to HFS and in some cases, constantly during the entire experimental protocol. Results for both washout (10 to 20 minutes following high-frequency stimulation) and constant perfusion conditions were pooled together for statistic analysis since no difference was observed between these conditions. As in control conditions, enhancement of EPSCs in APV-treated slices became stable and long lasting after short-term PTP (see Figure 8(a)). The average normalized value of peak EPSCs in the presence of APV increased by a factor of 1.695 ± 0.145 and 1.707 ± 0.19 of baseline (pre-HFS) values at 30 and 50 minutes, respectively, values that were 1 significantly different from baseline values (*n* = 10, *P* < .01) and 2 not significantly different from increases found in control conditions (*P* > .05, see Figure 8(b)). In an additional two experiments, we also tested the effects of a higher concentration of APV (100 *μ*M), but both the induction and expression of LTP were still unaffected (data not shown).

**Figure 9:**
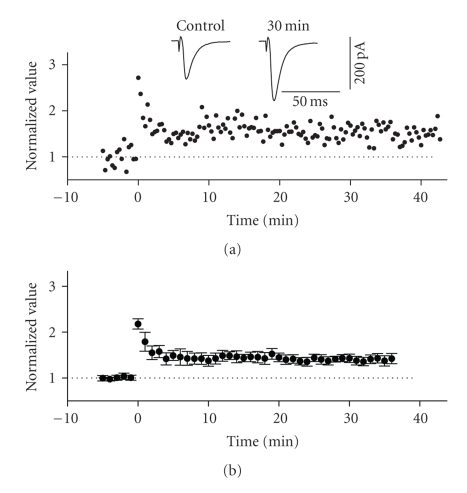
Induction of LTP in the ascending pathway was not blocked by sequestering of free postsynaptic calcium. (a) Normalized scaled amplitude of peak EPSCs evoked by test pulses in a layer II cell loaded with 10 mM BAPTA is plotted against time. HFS was routinely applied about 15 minutes after formation of whole-cell mode to ensure adequate BAPTA diffusion. The inset shows two traces reflecting averaged EPSCs at baseline (pre-HFS) and at 30 minutes post-HFS. HFS-induced enhancement of EPSCs was still observed. (b) Average normalized scaled amplitude of peak EPSCs across 5 BAPTA-loaded cells. HFS at time zero induced both short-term (PTP) and long-term potentiation. The value at 30 minutes post-HFS (1.427 ± 0.092) was slightly reduced compared to control conditions but significantly different from baseline (*P* < .05).

Given that calcium flux arising from activation of either postsynaptic calcium channels or metabotropic glutamate receptors can also induce synaptic plasticity independently of NMDA receptor activation, we tested whether fast chelation of free intracellular calcium with BAPTA would block LTP in this pathway. High-frequency stimulation was applied 15 minutes after whole cell configuration to allow for full intracellular dialysis of BAPTA to occur. With 10 mM BAPTA in the pipette solution, the amplitude of LTP was markedly reduced although PTP was consistently induced. Under this condition (see Figure 9(a)), the average increase in the normalized peak value of EPSCs was only 1.427 ± 0.092 30 minutes post-HFS, a value reflecting a significant reduction (to 86%) of synaptic enhancement from control experiments (see Figure 9(b); *n* = 5). When we included 20 mM BAPTA in the recording pipette we could completely abolish LTP (but not PTP: *n* = 7). However, this manipulation also significantly decreased the input resistance of the postsynaptic cell by an average of 20% (data not shown).

The above data, though not completely conclusive, suggest that postsynaptic calcium entry may partially play a role in LTP induction in the layer II-II pathway. Since NMDA receptor-channels were obviously not the source of this calcium, we then explored two other possibilities: (1) calcium entry via voltage-gated Ca^2+^ channels and (2) calcium entry via metabotropic glutamate receptors (mGluRs). To assess the first possibility we attempted to induce LTP by pairing low-frequency presynaptic stimulation (0.05–01 HZ) with large amplitude postsynaptic depolarizing voltage-clamp steps (200 milliseconds at −20 mV) which have been shown to elicit LTP in other systems. However, this protocol did not cause any significant change in the amplitude of the baseline EPSC (not shown, *P* > .05, *n* = 5). To assess the second possibility, we blocked mGluRs with the broad spectrum antagonist MCPG (100 *μ*M). However, in 5 neurons tested, HFS continued to induce LTP to a similar extent as in control conditions (not shown).

**Figure 10:**
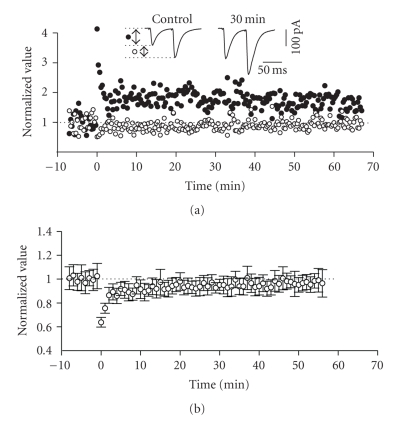
Expression of LTP in the ascending pathway was associated with a slight change in PPF ratio. (a) Normalized scaled amplitude of the peak EPSCs evoked by the first of a pair of test pulses separated by 60 milliseconds (filled circles), and the normalized PPF ratio (open circles) in a layer II cell is plotted against time. The inset shows two traces reflecting averaged paired EPSCs at baseline (pre-HFS) and at 30 minutes post-HFS. PPF ratio was calculated as the amplitude of the peak of the second EPSC divided by the first. (b) Average normalized scaled amplitude of PPF ratio in 8 cells. HFS at time zero induced a short-term decrease of PPF which decayed back to near baseline levels. The values at 30 and 50 minutes after HFS, however, (0.917 ± 0.084 and 0.938 ± 0.062, resp.) were significantly different from baseline (*P* < .01).

Previous findings using field potential recordings [22] have shown that LTP in the ascending layer V to layer associative pathway of the EC is partially NMDA-independent. By measuring changes in paired-pulse facilitation (PPF), these researchers suggested that this non-NMDA component of LTP reflected an increase in presynaptic transmitter release. To assess whether LTP in the layer III input to layer II cells was expressed presynaptically, we computed changes in the PPF ratio before and after expression of LTP. PPF from the intrinsic ascending layer III pathway was observed in 77% cells (17 of 23 cells) during control (pre-HFS) conditions in the presence of 2.4 mM extracellular Ca^2+^. The range of ratios observed was between 1.08 and 1.96, with an average value of 1.36 ± 0.081. Only cells displaying both PPF and LTP were examined (see Figure 10(a)). Directly following (1 minute post) HFS (i.e., during the expression of PTP) the normalized PPF ratio was reduced by a factor of 0.64 ± 0.048 compared to baseline values (*n* = 10, see Figure 10(b)). This decrease, however, was only transient since PPF values showed an initial quick and later slow increase which brought them back to near baseline (unity) values (see Figure 10(b)). The average normalized PPF ratio measured 30 and 50 minutes after application of HFS was 0.944 ± 0.065 and 0.966 ± 0.068, respectively, which were only slightly, but significantly, different from that during baseline period (*P* < .01). Thus, it would appear that only a small component of the LTP expressed in this pathway reflected an increase in neurotransmitter release.

### 3.5. Molecular mechanistic differences in LTP of the horizontal and columnar associative pathways

**Figure 11:**
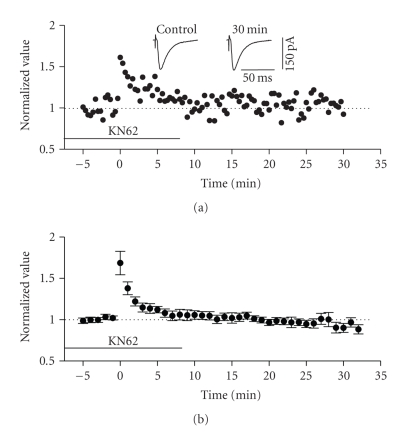
Induction of LTP in the horizontal pathway required intact CaMKII signalling. (a) Normalized scaled amplitude of peak EPSCs evoked by test pulses in a layer II cell is plotted against time. Time point of application of 3.8 *μ*M KN62 is indicated by the line. HFS at time zero led to a slight short-term potentiation (PTP) but no long-term potentiation. The inset shows two traces reflecting averaged EPSCs at baseline (pre-HFS) and at 30 minutes post-HFS. (b) Average normalized scaled amplitude of peak EPSCs across 6 cells in the presence of KN62 as indicated by the line. The value at 30 minutes post-HFS (0.8998 ± 0.0545) was significantly reduced from baseline (*P* < .05).

**Figure 12:**
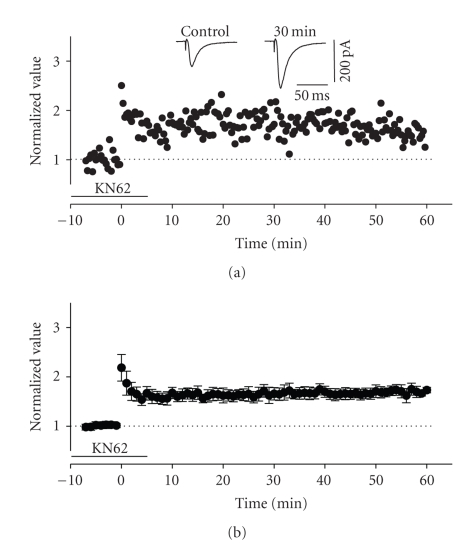
Induction of LTP in the ascending pathway was not dependent upon intact CaMKII signalling. (a) Normalized scaled amplitude of peak EPSCs evoked by test pulses in a layer II cell is plotted against time. Time point of application of 3.8 *μ*M KN62 is indicated by the line. HFS led to only a slight short-term potentiation (PTP) but a prominent long-term potentiation. The inset shows two traces reflecting averaged EPSCs at baseline (pre-HFS) and at 30 minutes post-HFS. (b) Average normalized scaled amplitude of peak EPSCs across 10 cells in the presence of KN62 as indicated by the line. The value 30 minutes after HFS was 1.656 ± 0.124 which was significantly different from baseline values (*P* < .01) and no different from LTP in control conditions (*P* > .05).

Based on the differences in induction properties of LTP in the two pathways examined, we sought to evaluate their potential differential dependence on independent second-messenger pathways. We first tested the effect of the widely-used CamKII specific inhibitor, KN62. KN62 (3.8 *μ*M) was applied to slices though bath perfusion for more than 10 minutes before HFS was delivered. This compound had differential effects on LTP expressed in the horizontal versus the columnar associative pathways. HFS of layer I in the presence of KN62 failed to elicit LTP in layer II cells (see Figure 11). The normalized average of EPSC amplitude at 30 minutes post-HFS was 0.8998 ± 0.0545, which was actually significantly reduced with respect to baseline values (*n* = 6, *P* < .05). In contrast, HFS of layer III in the presence of KN62 was still able to produce LTP in all layer II cells tested (see Figure 12), although in two cells it did suppress the expression of PTP. The normalized average of EPSC amplitude at 30 minutes after HFS was 1.656 ± 0.124, a value which reflected a significant facilitation (*n* = 11, *P* < .01) and which was not significantly different from the degree of potentiation expressed in control conditions (unpaired *t*-test, *P* > .05).

**Figure 13:**
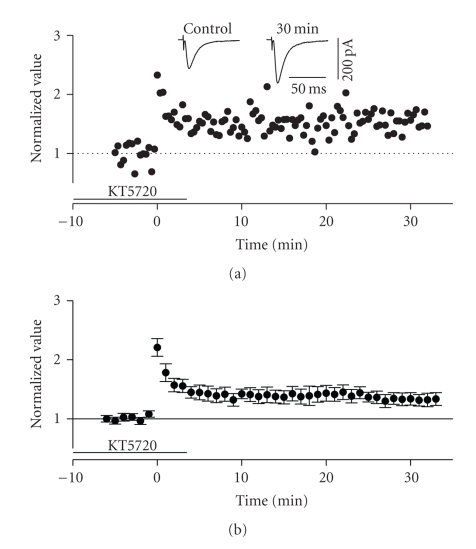
Induction of LTP in the ascending pathway was partially dependent upon intact PKA signalling. (a) Normalized scaled amplitude of peak EPSCs evoked by test pulses in a layer II cell is plotted against time. Time point of application of 250 nM KT5720 is indicated by the line. HFS led to intact short-term potentiation (PTP) but the degree of long-term potentiation was slightly decreased although still intact. The inset shows two traces reflecting averaged EPSCs at baseline (pre-HFS) and at 30 minutes post-HFS. (b) Average normalized scaled amplitude of peak EPSCs across 10 cells in the presence of KT5720 as indicated by the line. The value 30 minutes after HFS was 1.338 ± 1.016 which was significantly different from baseline values (*P* < .01) but was also significantly reduced in comparison to control conditions (*P* < .01).

Since LTP in the ascending pathway did not appear to be dependent on CaMKII, we next tested its dependence on PKA signalling. To inhibit PKA activity, we used bath applications of the catalytic subunit inhibitor KT5720 (250 nM). This manipulation, applied for 10 minutes before HFS, did not completely block LTP in layer II cells but did significantly suppress it (see Figure 13). The normalized average of EPSC amplitude at 30 minutes post-HFS was 1.338 ± 1.016 (*n* = 8) which was significantly smaller than that shown in control conditions (unpaired *t*-test, *P* < .01). Thus, at least part of the LTP expressed in this pathway appears to depend upon PKA signalling.

## 4. DISCUSSION

In this study, we investigated the basic characteristics of synaptic communication and plasticity in two independent pathways converging on EC layer II principal neurons, the horizontal input (containing cortical afferents and intralaminar associative connections between cells of layer II), and the ascending columnar (interlaminar) layer III input, respectively. Although prior work has been conducted on the properties of LTP in the superficial layers of the EC, few have focussed on cellular mechanisms and none has compared the superficial associative pathways that converge upon layer II. Indeed, a recent whole-cell investigation of plasticity in EC layer II stellates concluded that only LTD is present in conditions similar to those reported here [28].

Our results demonstrated that both associational pathways within the EC were independent and had basic physiological and pharmacological characteristics similar to other glutamatergic synapses in the central nervous system. In addition, both displayed robust LTP evoked by HFS that was accompanied by small decreases in paired pulse facilitation ratio. Importantly, we confirmed that the induction mechanisms of LTP in these two convergent pathways demonstrated some important differences in terms of their dependence upon NMDA receptors and on their dependence upon postsynaptic calcium. This difference may reflect a differential dependence on second-messenger systems. While this is not the first direct demonstration of differential plasticity in independent pathways that terminate in the superficial layers of the EC, it is the first cellular investigation of this type. This confirms that the intrinsic circuit dynamics of the EC are highly complex.

### 4.1. Physiology of the horizontal and ascending excitatory inputs to EC layer II

The projection cells of EC layer II are important components of the medial temporal lobe memory circuit since they receive highly processed neocortical information and provide the hippocampus with its most massive input via the perforant path. Through feedforward excitatory (and presumably inhibitory) collaterals, these cells also form part of a rich autoassociative network [29] whereby neighbouring neurons can influence and entrain each other [30]. As well, the activity of these cells is influenced by inputs which derive from laminae located deeper to it [1, 2, 31, 32]. The functional significance of convergent horizontal and ascending pathways in layer II is unclear although it could be relevant in order to compare the results of processing in deeper layers to that conducted in the most superficial.

We have demonstrated in the present study that excitatory feedforward interlaminar communication is not only a feature of the traditional hippocampal input and output laminae of the EC (i.e., the most deep and the most superficial) but is also a feature of the independent superficial laminae (III and II) which comprise the cells of origin of the temporal ammonic and perforant pathways, respectively. As well, we demonstrated that both the horizontal and ascending associative pathways have properties consistent with excitatory glutamatergic synapses at a variety of locations in the central nervous system. Both demonstrated NMDA and non-NMDA (presumably AMPA) components. Indeed, pharmacological manipulations using both APV and CNQX completely abolished excitatory synaptic responses.

Also similar to other glutamatergic synapses throughout the nervous system, both the horizontal and ascending associative pathways exhibited LTP. These results are in stark contrast to those of Deng and Lei [28] who failed to show LTP in either the horizontal or ascending (layer V) inputs to EC layer II stellate cells using a variety of stimulation paradigms including HFS. The only apparent differences between our conditions was their inclusion of QX-314 (0.2 mM) in the whole-cell pipette (in order to suppress action potential generation) and their baseline periods used (>30 minutes) in order to attain stable series resistances following whole cell dialysis. Although we found that complete whole-cell dialysis over this time periods greater than 30 minutes appeared to negatively affect the probability of achieving LTP, Deng and Lei were also unable to demonstrate LTP using perforated patch methods. Thus, whole-cell washout is presumably not the cause of the discrepancy. However, we do note that both field and sharp-electrode intracellular recording methods have consistently demonstrated LTP in the superficial EC [21, 22, 27, 33–35]. Interestingly, Alonso et al. [27] suggested that the intrinsic rhythmic properties of EC layer II stellates, brought about by the interaction of I_h_ and I_Nap_ [25], may play a pivotal role in the induction of plasticity of afferent inputs. Since QX-314 blocks both these currents and suppresses resonant and oscillatory behavior [36], this manipulation may indeed be relevant.

We did not test if the expression of LTP in the layer I input was NMDA or non-NMDA dependent [33, 35] although with continued perfusions of APV in some experiments we continued to observe LTP in the layer III pathway, showing that it is presumably mediated by non-NMDA glutamate receptors.

### 4.2. Differential plasticity of the horizontal and ascending excitatory inputs to EC layer II

Although both pathways demonstrated intact NMDA receptor mediated transmission, only the induction of LTP in the horizontal pathway required NMDA signalling. As well, the calcium dependence of LTP in the layer III to II inputs did not appear to be as critical as that in the horizontal pathway. This suggests that the second-messenger systems mediating LTP in the two different input pathways are quite different and potentially independent. This makes layer II cells similar to other CNS neurons (such as CA3 pyramids in the hippocampus) in which different afferent inputs demonstrate NMDA- dependent and -independent LTP simultaneously [18]. A major difference is that the bulk of the LTP in EC layer II neurons in both pathways appears to be expressed via postsynaptic changes. Similar differences have been observed in LTP mechanisms in early postnatal (<P9) cortex and hippocampus [37, 38]. However, this is the first report to our knowledge showing this kind of difference in two independent pathways to the same sets of neurons in adult (>P27) brain.

## References

[1] Amaral DG, Witter MP, Paxinos G (1995). Hippocampal formation. *The Rat Nervous System*.

[2] Witter MP, Wouterlood FG, Naber PA, van Haeften T (2000). Anatomical organization of the parahippocampal-hippocampal network. *Annals of the New York Academy of Sciences*.

[3] Eichenbaum H (2000). A cortical-hippocampal system for declarative memory. *Nature Reviews Neuroscience*.

[4] Schwarcz R, Witter MP (2002). Memory impairment in temporal lobe epilepsy: the role of entorhinal lesions. *Epilepsy Research*.

[5] Hyman BT, Van Hoesen GW, Damasio AR, Barnes CL (1984). Alzheimer's disease: cell-specific pathology isolates the hippocampal formation. *Science*.

[6] Van Hoesen GW, Augustinack JC, Dierking J, Redman SJ, Thangavel R (2000). The parahippocampal gyrus in Alzheimer's disease. Clinical and preclinical neuroanatomical correlates. *Annals of the New York Academy of Sciences*.

[7] Zhou W, Jiang D, Raisman G, Zhou C (1998). Embryonic entorhinal transplants partially ameliorate the deficits in spatial memory in adult rats with entorhinal cortex lesions. *Brain Research*.

[8] Young BJ, Otto T, Fox GD, Eichenbaum H (1997). Memory representation within the parahippocampal region. *The Journal of Neuroscience*.

[9] Suzuki WA, Eichenbaum H (2000). The neurophysiology of memory. *Annals of the New York Academy of Sciences*.

[10] Egorov AV, Hamam BN, Fransén E, Hasselmo ME, Alonso A (2002). Graded persistent activity in entorhinal cortex neurons. *Nature*.

[11] Bliss TVP, Collingridge GL (1993). A synaptic model of memory: long-term potentiation in the hippocampus. *Nature*.

[12] Gu JG, Albuquerque C, Lee CJ, MacDermott AB (1996). Synaptic strengthening through activation of Ca^2+^-permeable AMPA receptors. *Nature*.

[13] Grover LM, Teyler TJ (1990). Two components of long-term potentiation induced by different patterns of afferent activation. *Nature*.

[14] Grover LM, Teyler TJ (1995). Different mechanisms may be required for maintenance of NMDA receptor-dependent and independent 
forms of long-term potentiation. *Synapse*.

[15] Malenka RC, Bear MF (2004). LTP and LTD: an embarrassment of riches. *Neuron*.

[16] Nguyen PV, Woo NH (2003). Regulation of hippocampal synaptic plasticity by cyclic AMP-dependent protein kinases. *Progress in Neurobiology*.

[17] Nicoll RA, Malenka RC (1995). Contrasting properties of two forms of long-term potentiation in the hippocampus. *Nature*.

[18] Zalutsky RA, Nicoll RA (1990). Comparison of two forms of long-term potentiation in single hippocampal neurons. *Science*.

[19] Katsuki H, Kaneko S, Tajima A, Satoh M (1991). Separate mechanisms of long-term potentiation in two input systems to CA3 pyramidal 
neurons of rat hippocampal slices as revealed by the whole-cell patch-clamp technique. *Neuroscience Research*.

[20] Weisskopf MG, Castillo PE, Zalutsky RA, Nicoll RA (1994). Mediation of hippocampal mossy fiber long-term potentiation by cyclic AMP. *Science*.

[21] Yun SH, Mook-Jung I, Jung MW (2002). Variation in effective stimulus patterns for induction of long-term potentiation across 
different layers of rat entorhinal cortex. *The Journal of Neuroscience*.

[22] Yang S, Lee DS, Chung CH, Cheong MY, Lee C-J, Jung MW (2004). Long-term synaptic plasticity in deep layer-originated associational projections to 
superficial layers of rat entorhinal cortex. *Neuroscience*.

[23] Ma L, Dickson CT, Alonso A (1999). Long-term potentiation in the intrinsic ascending pathways of the entorhinal cortex. *Society for Neuroscience Abstracts*.

[24] Alonso A, Klink R (1993). Differential electroresponsiveness of stellate and pyramidal-like 
cells of medial entorhinal cortex layer II. *Journal of Neurophysiology*.

[25] Dickson CT, Magistretti J, Shalinsky MH, Fransén E, Hasselmo ME, Alonso A (2000). Properties and role of *I*
_*h*_ in the pacing of subthreshold 
oscillations in entorhinal cortex layer II neurons. *Journal of Neurophysiology*.

[26] Blanton MG, Lo Turco JJ, Kriegstein AR (1989). Whole cell recording from neurons in slices of reptilian and mammalian cerebral cortex. *Journal of Neuroscience Methods*.

[27] Alonso A, de Curtis M, Llinás RR (1990). Postsynaptic Hebbian and non-Hebbian long-term potentiation of synaptic efficacy in the entorhinal cortex in slices and in 
the isolated adult guinea pig brain. *Proceedings of the National Academy of Sciences of the United States of America*.

[28] Deng P-Y, Lei S (2007). Long-term depression in identified stellate neurons of juvenile rat entorhinal cortex. *Journal of Neurophysiology*.

[29] Klink R, Alonso A (1997). Morphological characteristics of layer II projection neurons in the rat medial entorhinal cortex. *Hippocampus*.

[30] Dickson CT, Biella G, de Curtis M (2000). Evidence for spatial modules mediated by temporal synchronization of 
carbachol-induced gamma rhythm in medial entorhinal cortex. *The Journal of Neuroscience*.

[31] Dickson CT, Alonso A (1997). Muscarinic induction of synchronous population activity in the entorhinal cortex. *The Journal of Neuroscience*.

[32] Kloosterman F, van Haeften T, Witter MP, Lopes da Silva FH (2003). Electrophysiological characterization of interlaminar entorhinal connections: an essential link for 
re-entrance in the hippocampal-entorhinal system. *European Journal of Neuroscience*.

[33] de Curtis M, Llinás RR (1993). Entorhinal cortex long-term potentiation evoked by theta-patterned stimulation of 
associative fibers in the isolated in vitro guinea pig brain. *Brain Research*.

[34] Jung H-Y, Mickus T, Spruston N (1997). Prolonged sodium channel inactivation contributes to dendritic 
action potential attenuation in hippocampal pyramidal neurons. *The Journal of Neuroscience*.

[35] Yun SH, Huh K, Jung MW (2000). Selective enhancement of non-NMDA receptor-mediated responses following induction 
of long-term potentiation in entorhinal cortex. *Synapse*.

[36] Hutcheon B, Miura RM, Puil E (1996). Subthreshold membrane resonance in neocortical neurons. *Journal of Neurophysiology*.

[37] Kirkwood A, Silva A, Bear MF (1997). Age-dependent decrease of synaptic plasticity in the 
neocortex of *α*CaMKII mutant mice. *Proceedings of the National Academy of Sciences of the United States of America*.

[38] Yasuda H, Barth AL, Stellwagen D, Malenka RC (2003). A developmental switch in the signaling cascades for LTP induction. *Nature Neuroscience*.

